# Value‐Based Model for Vascular Access Management in the End‐Stage Kidney Disease Population

**DOI:** 10.1111/sdi.70024

**Published:** 2026-04-09

**Authors:** Daniel Raskin, Tushar J. Vachharajani, Sasan Partovi, Abdullah Khan, Sean P. Lyden, Levester Kirksey

**Affiliations:** ^1^ Interventional Radiology Division Cleveland Clinic Cleveland Ohio USA; ^2^ Vascular Surgery, Heart, Vascular and Thoracic Institute Cleveland Clinic Cleveland Ohio USA; ^3^ Department of Medicine John D Dingell Veterans Affairs Medical Centre Detroit Michigan USA; ^4^ Wayne State University School of Medicine Detroit Michigan USA

**Keywords:** arteriovenous fistula, dialysis economics, hemodialysis, value‐based medicine, vascular access

## Abstract

**Background:**

Value‐based medicine (VBM) seeks to maximize patient‐relevant outcomes per unit cost. In end‐stage kidney disease (ESKD), vascular access (VA) is a dominant, modifiable driver of morbidity, mortality, and expenditure.

**Methods:**

We performed a narrative review of published studies and gray literature on VA creation, maintenance, and salvage in ESKD, focusing on clinical outcomes, patient experience, and economic impact. Findings were synthesized within a VBM framework relevant to clinicians, health‐system leaders, and policymakers.

**Results:**

Contemporary data confirm that tunneled dialysis catheters (TDCs) are associated with high rates of bloodstream infection, central venous injury, and mortality, and substantially higher costs than autogenous access. Arteriovenous fistulas (AVFs) offer the best long‐term value when creation is risk‐based, maturation is supported, and the access is actually used; nonmaturation, nonuse, and prolonged catheter dependence erode this advantage. Endovascular AVF and external support devices improve technical success and early patency but have uncertain cost‐effectiveness at current device prices. Arteriovenous grafts (AVGs) can provide greater net value than AVFs in selected patients (older, frail, or with poor veins) by shortening catheter exposure, at the expense of higher reintervention rates and maintenance costs. Across access types, circuit failure and recurrent interventions drive a substantial share of hemodialysis admissions and Medicare spending. Selective preoperative imaging, targeted duplex ultrasound in response to clinical findings, and ultrasound‐guided cannulation can improve access selection, maturation, and salvage while avoiding low‐value routine surveillance. Peritoneal dialysis remains underutilized despite comparable outcomes in many cohorts and lower average per‐patient costs than in‐center hemodialysis. Site‐of‐service optimization (office‐based/ASC vs. hospital) and multidisciplinary, life‐plan‐based access programs further reduce admissions, catheter days, and per‐patient costs.

**Conclusions:**

A value‐based VA strategy for ESKD should prioritize minimizing catheter exposure, tailoring AVF versus AVG use to patient risk, integrating PD where feasible, using selective imaging and monitoring, matching site of service to case complexity, and organizing multidisciplinary access teams aligned with quality and cost metrics.

**Abbreviations**
AVF: arteriovenous fistula
AVG: arteriovenous graft
ESKD: end‐stage kidney disease
IHD: intermittent hemodialysis
PD: peritoneal dialysis
RRT: renal replacement therapy
TDC: tunneled dialysis catheter
VBM: value‐based medicine
VA: vascular access

## 1 Introduction

Value‐based medicine (VBM) provides a systematic approach to maximizing outcomes that matter to patients relative to the healthcare resources consumed. The concept emerged from Berwick's “Triple Aim” of better patient experience, improved population health, and lower per‐capita cost [1].

End‐stage kidney disease (ESKD) is rapidly growing because of the increasing prevalence of cardio‐metabolic disorders such as diabetes, obesity, and hypertension, which are significant drivers of kidney disease, both independently and collectively. The associated care of this population of patients is chronic, complex, and resource‐intensive. Although healthcare payer models vary across the United States, United Kingdom, Canada, and OECD/EU, the economic pressures are largely borne by governmental insurance payers (publicly funded by general taxation). Thus, the topic is germane for providers and administrators globally.

The creation and maintenance of vascular access (VA) for intermittent hemodialysis (IHD) lack uniform standards and expected clinical outcomes. This results in high procedural volumes, broad variation in patterns of clinical care, diminished patient well‐being, and rising healthcare costs largely incurred by governmental insurance payers [2, 3]. While economic examples primarily reference US payment models and datasets, the value framework prioritizing clinical outcomes, patient‐centered metrics, and cost stewardship is broadly applicable across health systems where chronic kidney disease affects ~9% of the world's population (≈700 million people) and contributed to > 1.2 million deaths in 2017, with the fastest growth and largest unmet need in low‐ and middle‐income regions [4, 5].

This narrative review synthesizes current evidence and proposes an actionable VBM framework and care process for VA management that clinicians, health‐system leaders, and policymakers can apply to improve clinical quality with evidence‐based interventions that maximize outcomes while containing cost.

## 2 Economic and Policy Context

The current financial landscape of ESKD care represents the byproduct of the enduring need to control expenditures associated with a growing disease prevalence while concurrently incentivizing desired clinical outcomes. According to the 2024 US Renal Data System (USRDS) Annual Data Report, Medicare spent USD 48.9 billion on dialysis care in 2022: some 7% of the program's total budget while serving < 1% of beneficiaries [6]. The expenditure for ESKD remains at this level for two consecutive years ($51 billion in 2021), highlighting a persistent macroeconomic burden [6].

Two landmark federal initiatives created today's payment landscape. The 1972 Social Security Amendments extended Medicare entitlement to all citizens with ESKD, removing cost barriers but committing the program to open‐ended expenditure [7]. The 2011 End‐Stage Renal Disease Prospective Payment System (PPS) bundled dialysis‐related drugs, laboratory tests, and selected vascular access services into a single facility payment, encouraging efficiency but raising concern about under‐provision of non‐reimbursed care [8].

Quality and value are now nudged through the End‐Stage Renal Disease Quality Incentive Program (QIP), which withholds up to 2% of PPS payments from facilities that fail benchmarks such as catheter prevalence and infection rates [9]. In 2019, the Advancing American Kidney Health (AAKH) initiative and associated payment models set ambitious targets to reduce the incidence of kidney failure and to have 80% of incident patients treated with home dialysis or transplantation by 2025, reshaping incentives around modality choice and site of care [10].

## 3 Challenges in ESKD Management

The prevalence of ESKD in the United States and globally is rising, driven by increasing prevalence of cardio‐metabolic diseases, improved management of chronic medical conditions, and increased life expectancy [11, 12]. The number of individuals receiving treatment for ESKD increased from approximately 209,000 in 1991 to over 746,000 in 2018. This represents a more than threefold increase over nearly three decades, with a projected increase to 1,259,000 patients by 2030 [13].

An essential aspect of high‐quality care for ESKD patients involves the creation and sustained maintenance of functional vascular access (VA) to enable uninterrupted renal replacement therapy (RRT). Renal transplantation remains the gold standard therapy, offering clear advantages such as reduced infection rates, fewer unplanned hospital admissions, and lower kidney disease–related mortality. However, limited donor supply maintains the waiting list near 72,000, far below the number necessary to permit broad use of transplantation. Unfortunately, peritoneal dialysis remains a broadly underutilized modality for myriad reasons including, but not limited to, provider inertia [14]. As a result, intermittent hemodialysis (IHD) remains the primary RRT modality for about 70% of ESKD patients [15]. Reliable VA, autogenous arteriovenous fistula (AVF) or graft (AVG), reduces infection, unplanned emergency unit visit, hospital readmission, and access‐related mortality [16, 17].

Despite this knowledge, systemic lapses, delayed chronic‐kidney‐disease recognition, late nephrology referral, and lack of pre‐emptive VA creation collectively result in ≈80% of patients initiating dialysis with a tunneled dialysis catheter (TDC); moreover, 1 year later half remain catheter‐dependent [6]. Prolonged catheter exposure drives a pooled bloodstream‐infection incidence of 1.16 ± 0.70 per 1000 catheter‐days, with diabetes, coronary, and peripheral artery disease nearly doubling risk vis‐à‐vis autogenous access [18]. Each sepsis episode adds ≈$23,000 to hospital costs and compromises transplant eligibility [19].

In recognition of these issues, the updated 2019 KDOQI clinical practice guidelines for vascular access pivot away from the earlier “Fistula First” paradigm (championed by the Fistula First Breakthrough Initiative) and instead advocate an individualized ESKD Life‐Plan approach [16], which prioritizes the “best access” to reduce TDC exposure. This patient‐centric strategy balances all available options based on each patient's goals, life expectancy, comorbidities, and vascular anatomy. The shift acknowledges that a one‐size‐fits‐all approach to VA is not optimal.

**FIGURE 1:**
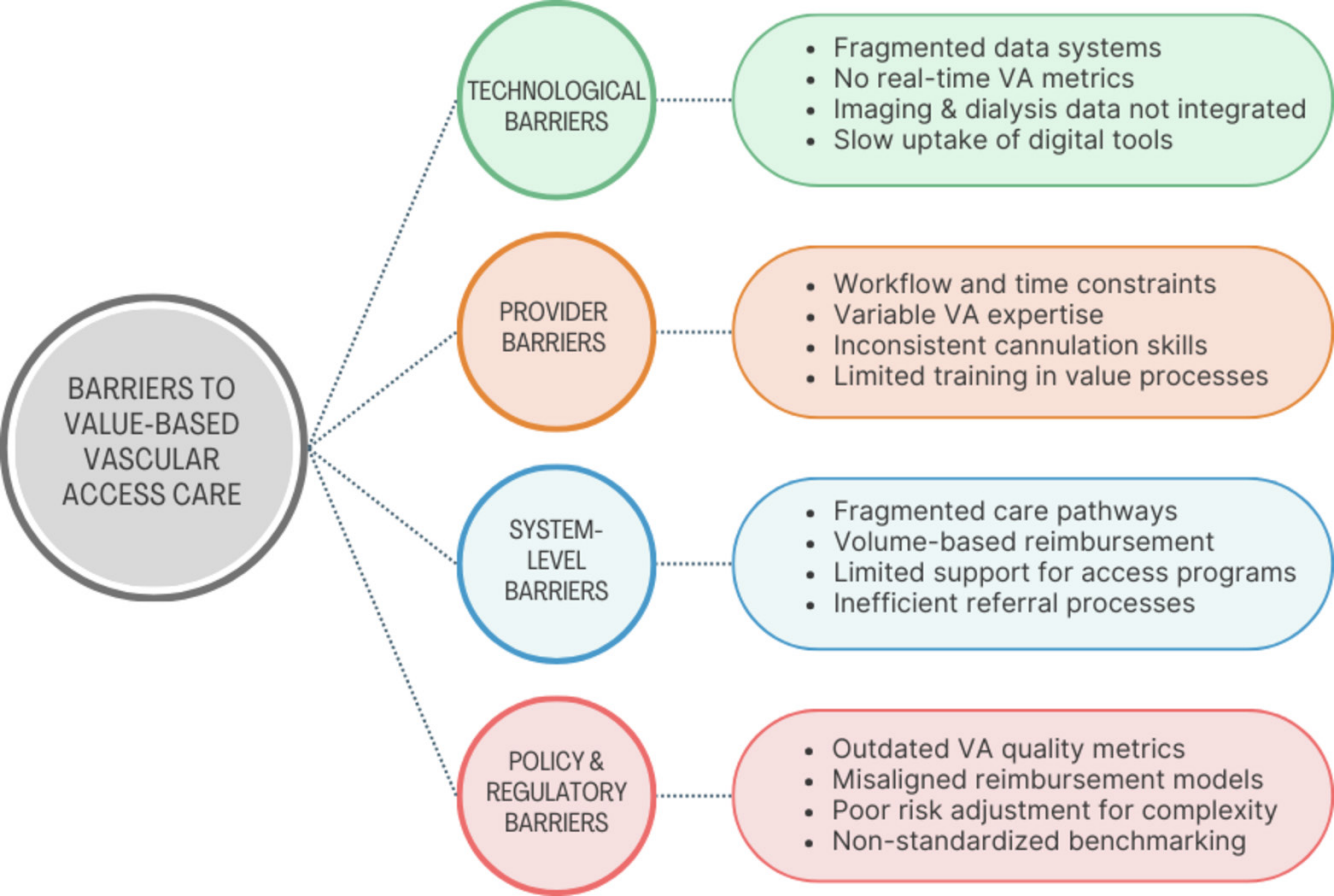
Schematic overview of key technological, provider, system‐level, and policy barriers that contribute to suboptimal vascular access outcomes in patients with ESKD. [Colour figure can be viewed at wileyonlinelibrary.com]

Major technological, provider‐related, system‐level, and policy obstacles contributing to these persistent issues are summarized in Figure 1.

## 4 Achieving Functional Access

### 4.1 Tunneled Dialysis Catheters

In the United States, TDC dependence is a major driver of morbidity, with vascular access complications accounting for roughly 12% of hemodialysis admissions nationwide and catheter‐related infection as the dominant cause [6, 20]. A 2024 multicenter prospective cohort (≈2100 patients; 198,000 catheter‐days) reported 0.78 catheter‐related bloodstream infection (CRBSI) episodes per 1000 catheter‐days, with risk highest during the first 90 days [21]. In a national stepped‐wedge, cluster‐randomized bundle intervention (REDUCCTION), tunneled catheter–related bloodstream infection rates were reduced by roughly one‐third, underscoring the value of standardized insertion and maintenance protocols for catheter care [22]. Broader US hospital data from the Agency for Healthcare Research and Quality place the average central‐line infection cost at a median $28,709 in vascular access costs per patient‐year versus $10,642 for peers who transition to an AVF [23]. A 2023 national stepped‐wedge cluster trial (9872 catheters; 1.12 million catheter‐days) reported 0.91 dysfunction‐driven removals per 1000 catheter‐days, essentially one flow‐limiting event every 1100 catheter‐days, and showed that evidence‐based insertion/maintenance bundles can lower this rate by one‐third [24]. Imaging studies demonstrate de‐novo central‐venous stenosis in 13% after a first TDC, and pooled cohorts show 20%–40% stenosis/occlusion when dwell time exceeds 6 months or the subclavian route is used [25, 26]. Even under modern ultrasound‐guided insertion, 2%–4% of TDC placements require immediate revision for malposition or inadequate flow [27]. Survival analyses reinforce the clinical penalty: Tunneled catheters confer a 2.8‐fold higher adjusted mortality hazard than AVFs over 7 years [28].

**Take home message:** TDCs deliver necessary short‐term access but carry a steep penalty in complications, cost, and survival, so their use and dwell time should be minimized whenever possible.

### 4.2 Creation of Permanent Dialysis Access

#### 4.2.1 Arteriovenous Fistulas (AVF)

AVFs remain the guideline‐preferred permanent access, yet nationwide utilization has slipped: The 2024 USRDS report shows the proportion of prevalent hemodialysis patients dialyzing via a fistula fell from 64% in 2017 to 59% in 2022 [6, 29]. During this interval, practice patterns evolved toward more individualized “patient‐first” and “catheter‐last” strategies, including broader use of AVGs in selected patients, and COVID‐19 related disruptions [30]. AVF nonmaturation remains an important barrier: Meta‐analysis places primary failure at 30%–70%, with 1‐year primary patency (patency of the index AVF without thrombosis or any reintervention on the access) averaging ~60% [31], and a 43,457‐patient Medicare cohort reports primary patency of 68% at 90 days but only 32% at 1 year [32]. When maturation stalls, catheter dependence lingers, eroding the value advantage of autogenous access.

Endovascular fistula platforms (endoAVF) now achieve technical success > 96% and primary patency 70% at 12 months, surpassing contemporary surgical radiocephalic AVF results [33]. Extravascular support devices have demonstrated improved AVF outcomes in a US pivotal study, including a 2‐year cumulative patency ~77%, with no device‐related serious adverse events; definitive comparative advantages over unsupported AVFs should be interpreted in light of evolving evidence [34].

Regarding site of service, office‐based or ambulatory surgical center pathways for AVF creation and maintenance have shown comparable outcomes with lower facility costs in appropriate settings, aligning with value‐based goals [35, 36]. Contemporary Medicare reimbursement benchmarks show substantially higher outpatient payments for endoAVF than open surgical AVF (reflecting device and facility costs, whereas AVG entails higher material costs and higher index‐year spend than AVF in all‐payer and Medicare cohorts). US cost‐effectiveness modeling suggests that endoAVF is not cost‐effective versus open AVF at current price differentials unless device costs fall or clear downstream clinical benefits (reduced TDC exposure/circuit reintervention rates) are realized [37]. In contrast, European modeling from the Italian National Health Service perspective suggests that endoAVF may be cost‐saving compared with surgical AVF in selected scenarios [38].

These differences reinforce evaluating incremental cost against absolute benefit and expected offsets [37, 39]. To avoid misinterpreting device value, incremental costs should be scaled to the absolute benefit rather than relative gains and netted against downstream offsets (e.g., fewer catheter‐days, infections, reinterventions, and visits) [6, 22, 23, 40, 41]. This framing is applied across endoAVF, external support devices, and adjuncts (stent grafts and drug‐coated balloons), aligning evaluation with patient‐relevant outcomes and contemporary US cost/payment models [33, 34, 42, 43, 44, 45].

Using total care expenditure/per patient circuit type, AVF demonstrates superiority to AVG once maturation is achieved. In a 12,716‐patient all‐payer study, AVF creation cost $9388 versus $13,539 for AVG in the index year, and multivariable modeling showed a $3557 lower cumulative 3‐year spend for AVFs. Similar Medicare analyses echo a $2000–$4000 annual saving per patient when AVFs mature successfully [46].

A key value consideration is the proportion of pre‐dialysis AVFs that are never used despite maturation. In a prospective Canadian cohort (*n* = 257), only 55% of created AVFs were ultimately used for dialysis when competing risks were accounted for, with ~17% of patients dying or changing modality before use. Nonuse and early failure carry measurable economic penalties: In a Medicare claims analysis, annualized vascular access costs in the 2.5 years after AVF creation were $31,630 (2013 USD) per patient‐year when the AVF was never used versus $7871 when primary patency was maintained, likely reflecting persistent catheter dependence and additional access procedures [47].

These data support replacing eGFR‐triggered referral with risk‐based timing using the Kidney Failure Risk Equation (KFRE). Integrating KFRE into a life‐plan rubric can reduce futile procedures, catheter dependence, and total cost while preserving patient‐centered timing. In population‐based analyses, adding a 2‐year KFRE threshold > 40% to standard referral increased on‐time AV access starts (49% → 58%) and reduced “too‐early” creation (31% → 18%); decision‐curve analyses in a national registry similarly favored KFRE > 40%–50% when the harm of unnecessary surgery is salient and showed that many patients who start dialysis with a catheter had exceeded KFRE thresholds ~9–10 months before kidney replacement therapy [47, 48, 49, 50, 51].

**Take‐home message:** AVFs are highest value when creation is risk‐based, maturation is supported, and the fistula is actually used; unnecessary or unused AVFs and high‐cost adjuncts without clear downstream benefit erode that value.

#### 4.2.2 Arteriovenous Grafts (AVGs)

Evidence accumulated over the past decade shows that selected cohorts: frail older adults, patients with limited life expectancy, and those with inadequate native veins may realize greater net value from primary AVGs than from fistulas [52]. Up‐front expenditure is higher: A 2023 systematic review of 33 economic studies reported a mean procedural cost of $4260 for AVG creation (range $640–$11,557) versus about $3400 for surgical fistula creation [53, 54]. AVGs can be cannulated within 2–3 weeks, often sparing months of catheter exposure, but their shorter patency and higher complication burden erode this early advantage. Contemporary registry data document ≈0.36 thrombosis‐or‐stenosis interventions per patient‐year for AVGs, nearly double the ≈0.18 recorded for fistulas, and first‐year access‐related spending averages $15,900 for grafts compared with $9800 for fistulas [39, 43].

The published patency associated with AVGs varies widely across studies, reflecting cohort heterogeneity and differences in follow‐up [3, 55, 56]. Because AVFs are prone to early nonmaturation, while AVGs more often fail later during the maintenance phase and require higher intervention rates [3, 57, 58], comparative studies that report intent‐to‐treat outcomes and incorporate catheter dependence, salvage procedures, and costs are needed to clarify the relative value of each access type.

**Take‐home message**: AVGs can offer higher net value than AVFs for carefully selected patients by shortening catheter time, but this benefit is offset by higher upfront and maintenance costs and a greater need for reinterventions.

## 5 Maintenance of the Dialysis Circuit

A functioning VA circuit is the driver for uninterrupted renal‐replacement therapy; once the circuit fails, emergency procedures, hospitalizations, and cost escalate. Contemporary USRDS surveillance shows that 48% of new fistulas lose primary unassisted patency within 2 years of dialysis start, confirming the steep attrition curve inherent to VA biology [6]. This inescapable natural history of the VA circuit drives high rates of reintervention, contributing to a substantial burden on both patients and the healthcare system [44].

Dysfunction translates directly into resource use. VA‐related problems account for more than 12% of all hemodialysis admissions across US centers, a proportion unchanged despite decade‐long payment reforms [20]. Each salvage procedure compounds expenditure: A systematic review of 33 economic studies reported a mean cost of $1300–$3900 per endovascular or open intervention, while office‐based practices still average some $3800 in annual maintenance charges per fistula [36]. Using Medicare claims, the calculated initial‐year creation and maintenance costs of $9400 for AVFs versus $13,500 for AVGs are reported, with grafts continuing to out‐spend fistulas by $1200–$1600 annually through Year 3 [46].

VA upkeep drains Medicare of well over half a billion dollars each year: Professional‐fee payments for fistula and graft maintenance reached $399 million in 2018, and total program outlays for vascular access procedures climbed to $531 million in 2020, despite the 4%–5% cut in per‐procedure reimbursement [45, 59].

A detailed comparison of clinical outcomes, infection risk, maintenance requirements, and economic burden for arteriovenous fistulas, grafts, and tunneled dialysis catheters is presented in Table 1.

**TABLE 1:** Access performance metrics for dialysis vascular access types.

Parameter	Arteriovenous fistulas (AVFs)	Arteriovenous grafts (AVGs)	Tunneled dialysis catheters (TDCs)
Usability time [16, 45]	6–12 weeks	2–4 weeks	Immediate
Primary failure rate [25, 30, 42]	30%–70%	~15%	≈2%–4%
12‐month access durability (AVF/AVG = primary patency; TDC = exchange‐free survival) [30, 42, 55]	≈60% primary patency	≈50% primary patency	≈30%–40% cumulative patency^a^
Infection rate (/1000 days) [7, 16, 18]	0.3 ± 0.15	0.8 ± 0.25	1.2 ± 0.7
Interventions per patient‐year [4, 16, 45]	0.2–1.0 (mostly endovascular)	1.0–2.0 (angioplasty, thrombectomy)	0.5–1.0 exchanges
Mean first‐year cost (USD) [38, 45]	$9000–$10,000	$15,000–$16,000	$20,000–$30,000
Annual maintenance cost (USD) [38, 39, 45]	$3800–$6800	$7000–$11,000	$13,625–$28,700
Procedural cost (Medicare, USD) [45, 52]	$3400–$3500	$4000–$4500	$900–$1800
Ideal patient profile [16, 17, 30, 45]	Younger/healthier, good vessels, > 1 yr. life	Poor veins, frail, limited life span	Immediate need/bridge, poor access sites

^a^ For TDCs, durability is best summarized by exchange‐free survival and adverse‐event rates (e.g., dysfunction‐driven removals, CRBSI); “primary patency” is not comparable to AVF/AVG. Catheter removal does not imply conversion to a permanent AV access.

**Take‐home message**: Circuit failure is common and expensive, driving repeat interventions, hospitalizations, and substantial spending.

## 6 Room for Improvement

### 6.1 Evidence‐Based Decision‐Making in Vascular Access

Evidence‐based literature, such as the KDOQI guidelines, provides a framework to ensure that decisions about care are based on the best scientific evidence available [16]. Poor compliance with the guidelines has been associated with increased VA complications and is linked to patient morbidity and mortality. In cases of delayed or erroneous planning of access, the frequent and prolonged use of central venous catheters poses increased risks of infection and death. Non‐compliance also leads to greater economic expenditure due to longer hospital stays, repeated interventions, and intensive management of complications [60, 61, 62, 63, 64, 65]. The impact of key evidence‐based strategies and interventions on clinical outcomes and economic value is summarized in Table 2.

**TABLE 2:** Impact of evidence‐based strategies and interventions.

Strategy/intervention	Key outcome/metric	Cost impact
Early AVF creation/timely referral [16, 17]	Reduces TDC days by 60%; halves BSI risk	+$2–$4 k savings/year
Multidisciplinary care (MDC) team [55, 56]	Lowers admissions, missed dialysis, improves access	~$1500–$3000/patient/year savings
Routine Doppler vessel mapping [16, 57]	Lowers primary AVF failure (esp. high‐risk cases)	Reduces reinterventions, cost‐effective
Dedicated vascular access center [36, 56, 58]	29%–62% reduction in access hospitalizations, missed sessions	Major cost and outcome benefit
Outpatient/ambulatory site for maintenance [35, 59]	Lower peri‐op complication, faster cannulation, shorter wait	$800–$2000 per episode savings

**Take‐home message:** Consistent use of KDOQI‐based, evidence‐driven pathways reduces catheter dependence, complications, and avoidable costs.

### 6.2 Site of Service: Hospital‐Based Versus Ambulatory Surgical Centers

Office‐based laboratories (OBLs), free‐standing vascular access centers, and Medicare‐certified ambulatory surgical centers (ASCs) are recognized as cost‐effective alternatives for VA procedures. Site of service should be matched to patient complexity and resource needs. For patients with significant comorbidity, complex anatomy, or anticipated need for advanced imaging, anesthesia, or postoperative monitoring, hospital‐based care is appropriate and often safer; apparent outcome differences frequently reflect case‐mix rather than an intrinsic disadvantage of the hospital setting. In a contemporary retrospective comparative series of 389 arteriovenous fistulas (2016–2020), perioperative complications were 1.6% in an OBL versus 6.4% in a hospital outpatient department, with higher maturation (93% vs. 54%) and a shorter time to first cannulation (52 vs. 98 days) [36]. Technical success, 1‐year patency, and reintervention rates were otherwise equivalent, suggesting that for straightforward anatomy and lower comorbidity, shifting care out of the hospital does not compromise outcomes. Given its retrospective, nonrandomized design within a single health system's access program, these findings are hypothesis‐generating and require confirmation in larger prospective or randomized studies [35]. Shorter waiting times, faster recovery, and increased convenience may contribute to higher patient satisfaction and improved follow‐up care compliance [66]. ASCs likewise maintain comparable AVF maturation and long‐term patency in appropriate, lower risk patients, both inpatient and outpatient settings [35, 67]. Moreover, ambulatory procedures can reduce the risk of hospital‐acquired infection and readmission [35, 68].

After CMS changes in CPT code bundling in 2017, per‐procedure physician payment decreased from $1073 to $1025 (−4.5%), yet professional fees for access maintenance still totaled $399 million in 2018 [45]. Recent Physician Fee Schedule and OPPS/ASC rules (2022–2026) explicitly seek to “align payment toward lower‐cost sites of service,” sustaining a 30%–40% reimbursement differential in favor of OBL/ASC settings for common angioplasty or thrombectomy [36, 40, 69].

For patients with significant comorbidity or complex anatomy, hospital‐based care is often necessary and appropriate given access to advanced imaging, anesthesia, and postoperative monitoring. This factor in patient selection must be considered when hospital‐based procedures have worse outcomes and poor functional status [70].

**Take‐home message:** Match site of service to case complexity; OBL/ASC work well for lower risk patients, while complex cases belong in hospital.

### 6.3 Preoperative Imaging

Preoperative Doppler ultrasound (DUS) for vessel mapping significantly lowers AVF primary failure rates by identifying optimal anastomosis sites and assessing vessel quality, particularly in cases at high risk of access failure. However, it is not recommended as a routine [16, 71, 72, 73]. Additionally, it is advisable to employ various imaging techniques to assess vessel suitability for VA creation, including DUS for peripheral vessels and venography for potential central vein occlusion, while considering the patient's clinical situation and remaining kidney function [16]. Within a value‐based framework, programs should incorporate DUS‐based planning as part of multidisciplinary, OBL‐enabled pathways when feasible. While the initial cost of preoperative imaging is not always reimbursed, it can ultimately be cost‐effective by reducing the need for additional imaging, invasive procedures, and lowering AVF failure risks [74].

**Take‐home message:** Selective, risk‐based preoperative imaging improves access selection and can be cost‐effective when targeted.

### 6.4 Follow‐Up Monitoring

To prevent VA flow dysfunction, it is advised to regularly monitor it for patency through clinical assessment by a knowledgeable and experienced health practitioner [16]. KDOQI emphasizes that clinical monitoring is first‐line because there is insufficient evidence to recommend routine imaging surveillance for stenosis to enhance VA patency [16].

KDOQI emphasizes clinical monitoring as first‐line and notes insufficient evidence to support routine AVF or AVG surveillance with DUS or other imaging beyond clinical assessment [16]. In patients with concerning clinical findings (suspected aneurysm/pseudoaneurysm, thrombus, or stenosis), targeted DUS can complement examination by characterizing anatomy and flow and help guide the need and timing for angiographic evaluation [71, 75, 76]. Ultrasound‐guided cannulation, particularly early after creation and in challenging anatomy, should be incorporated where available to reduce cannulation attempts, infiltrations/hematomas, and catheter dependence [16, 44].

Elective revisions of stenoses identified through DUS have demonstrated a reduction in the need for urgent thrombectomies. This approach to access management yields substantial financial benefits by reducing access‐related hospitalizations, procedures, and overall costs through the proactive detection and treatment of stenosis before thrombosis occurs [77]. While postoperative monitoring programs entail additional costs, this is minimal compared to the significant savings achieved by avoiding more expensive acute circuit salvage procedures [78, 79, 80]. Some international groups promote adding sonographic monitoring to clinical evaluation; where adopted, programs should track local value signals (e.g., fewer urgent thrombectomies, access‐related admissions, and catheter‐days) to confirm net benefit [65, 74].

High‐flow vascular access circuits are associated with adverse cardiac remodeling and the development of chronic diastolic heart failure, with or without an overt high‐output state. KDOQI provides general guidance to monitor circuits with volume flow in excess of 2 L/min. Further investigation is needed to distinguish access‐related effects from the cardiac remodeling repeatedly observed with intermittent hemodialysis itself (e.g., dialysis‐induced ischemia/stunning, progressive diastolic dysfunction, and LV mass changes). A prudent approach is at least annual vascular access duplex ultrasound and transthoracic echocardiography for the first 2 years after a high‐flow designation, with intensified surveillance if patient‐reported heart‐failure symptoms (assessed using a validated tool) or echocardiographic parameters worsen [16, 81, 82, 83].

**Take‐home message:** Clinical monitoring is first‐line; selective DUS and ultrasound‐guided cannulation support earlier, safer intervention and fewer urgent salvages.

### 6.5 Peritoneal Dialysis

Within a value‐based framework, modality selection is upstream to vascular access exposure. Peritoneal dialysis (PD) can be employed as first‐line therapy or as a bridge, thereby avoiding or deferring hemodialysis VA creation and reducing tunneled‐catheter exposure. PD preserves residual kidney function and is associated with fewer early hospitalizations and comparable or better early survival in some cohorts while supporting home‐based care and patient autonomy. US analyses generally show lower average annual Medicare expenditures for PD than for in‐center hemodialysis, although outcomes depend on patient selection and program supports [84]. Accordingly, early PD evaluation and urgent‐start pathways should be integrated into life‐plan access decisions to minimize unnecessary VA procedures and catheter days [14, 40, 84, 85, 86]. The average annual costs for hemodialysis were approximately $108,656, compared to $91,716 for PD (2017 USD) [87]. Higher IV dialysis drug costs in hemodialysis contribute to the significant cost disparity, even though they have declined recently [87]. Over a 10‐year period, the average cost for PD per patient was approximately $336,309 compared to $352,712 for hemodialysis, leading to a significant cost difference. Additionally, PD was found to be more cost‐effective, with a cost per quality‐adjusted life year (QALY) of $87,127, compared to $108,527/QALY for hemodialysis [84].

**Take‐home message:** When feasible, PD as first‐line or bridge therapy lowers catheter exposure, supports home care, and reduces long‐term costs.

### 6.6 Multidisciplinary Approach

Vascular access planning should follow a life‐plan rubric and be delivered by a multidisciplinary team including nephrologists, dialysis nurses, vascular access coordinators, vascular surgeons, interventionalists, and access sonographers [88, 89]. VA centers should aim to deliver team‐based, programmatic care guided by shared decision‐making. Successful models of team‐based care in other conditions such as stroke and cardiovascular disease consistently show improved clinical outcomes, better care coordination, and enhanced patient experience [90, 91, 92, 93, 94, 95].

In an earlier US study, a dedicated multidisciplinary access center reduced hospitalizations and missed treatments, with estimated annual savings of $300–$750 million for a 250,000‐patient hemodialysis population and lower per‐patient costs than standard care [94]. Scheduling and navigating appointments for surgical access evaluation, procedures, and follow‐up can impose significant burdens of time, transportation, and technology on patients facing high social determinants of health challenges. These delays may ultimately impact clinical outcomes [40].

**FIGURE 2:**
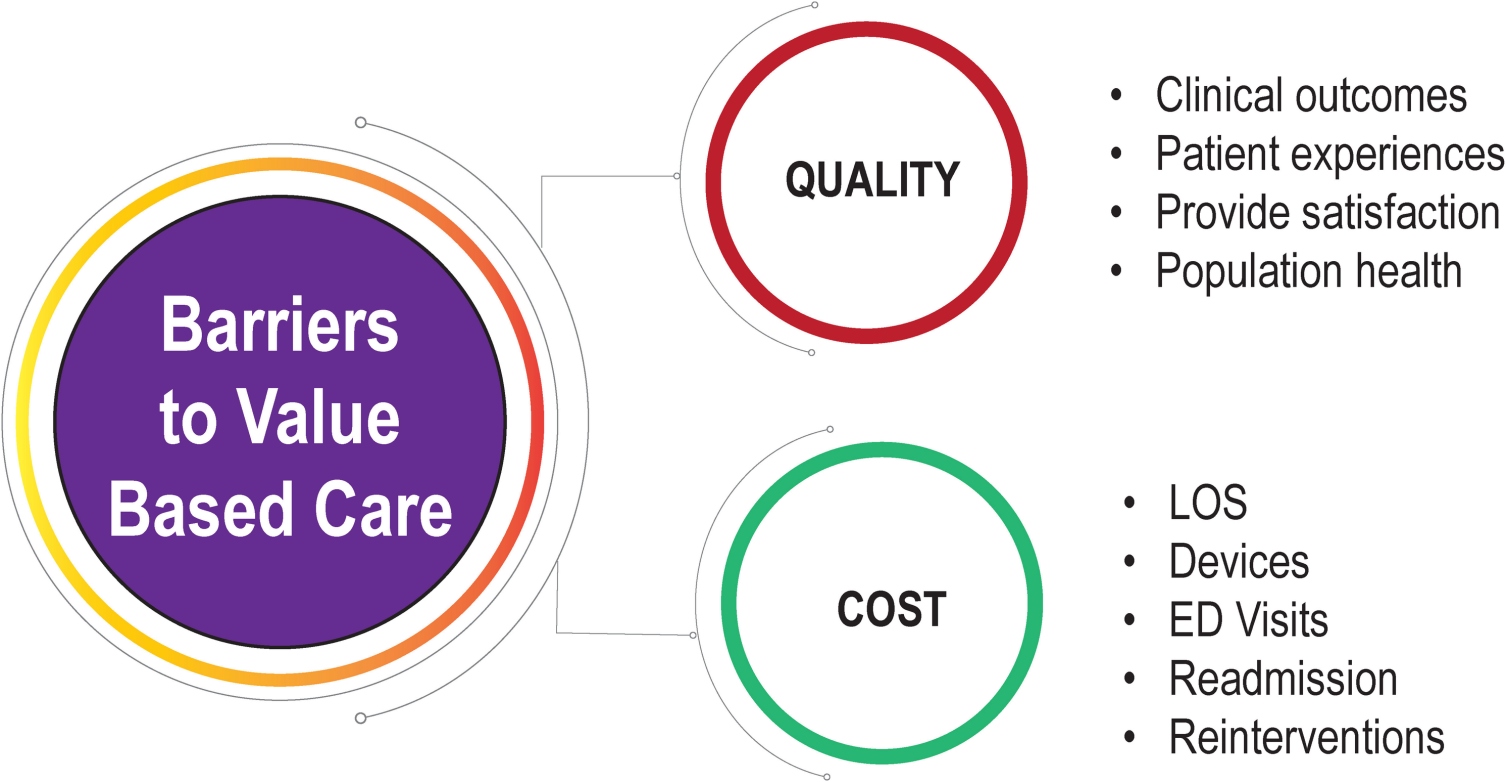
Illustration of a multidisciplinary, patient‐centered approach linking quality and cost domains in dialysis vascular management. [Colour figure can be viewed at wileyonlinelibrary.com]

Ideally, incentives should align with integrated practice units that deliver team‐based care with multidisciplinary teams working toward shared care goals, and shared returns tied to measurable quality metrics. Medicare's current quality incentive program is the “pay for performance,” value‐based purchasing model [96]. A simplified value framework that links key quality and cost domains in dialysis vascular management is depicted in Figure 2.

**Take‐home message:** Multidisciplinary, life plan–based access programs improve coordination, reduce admissions, and lower per‐patient costs.

## 7 Summary

The VA pathway remains a critical determinant of clinical and economic outcomes in ESKD care. While TDCs remain the most common initial access, they are associated with high infection rates, structural complications, and significantly higher costs compared to AVFs or AVGs. Despite guideline recommendations favoring early AVF placement, barriers including poor planning, complex anatomy, and variable provider practices contribute to delayed or inappropriate access selection.

This manuscript outlines the contemporary evidence on VA selection, maintenance, and surveillance from a VBM perspective. It emphasizes patient‐centered access planning, advances in endovascular fistula creation, cost comparisons between access types, and the growing role of outpatient settings in access care. Integration of multidisciplinary care teams, targeted imaging, and proactive monitoring strategies are highlighted as levers to improve outcomes while reducing total costs. Recognizing that one‐size‐fits‐all solutions are insufficient, this paper advocates for individualized, life plan–guided access decisions aligned with long‐term goals and resource stewardship.

## Funding

The authors have nothing to report.

## References

[1] D. M. Berwick , T. W. Nolan , and J. Whittington , “The Triple Aim: Care, Health, and Cost,” Health Affairs 27, no. 3 (2008): 759–769, 10.1377/hlthaff.27.3.759. 18474969

[2] A. K. Viecelli , E. O'Lone , B. Sautenet , et al., “Vascular Access Outcomes Reported in Maintenance Hemodialysis Trials: A Systematic Review,” American Journal of Kidney Diseases 71, no. 3 (2018): 382–391, 10.1053/j.ajkd.2017.09.018. 29203125

[3] K. J. Woodside , K. J. Repeck , P. Mukhopadhyay , et al., “Arteriovenous Vascular Access‐Related Procedural Burden Among Incident Hemodialysis Patients in the United States,” American Journal of Kidney Diseases 78, no. 3 (2021): 369–379.e1, 10.1053/j.ajkd.2021.01.019. 33857533 PMC8384666

[4] L. Wang , Y. He , C. Han , et al., “Global Burden of Chronic Kidney Disease and Risk Factors, 1990–2021: An Update From the Global Burden of Disease Study 2021,” Frontiers in Public Health 13 (2025): 1542329, 10.3389/fpubh.2025.1542329. 40843419 PMC12366504

[5] K. Xie , H. Cao , S. Ling , et al., “Global, Regional, and National Burden of Chronic Kidney Disease, 1990–2021: A Systematic Analysis for the Global Burden of Disease Study 2021,” Frontiers in Endocrinology 16 (2025): 1526482, 10.3389/fendo.2025.1526482. 40110544 PMC11919670

[6] United States Renal Data System , “2025 USRDS Annual Data Report,” National Institute of Diabetes and Digestive and Kidney Diseases, National Institutes of Health (2025), https://usrds‐adr.niddk.nih.gov/2025.

[7] R. A. Rettig , Origins of the Medicare Kidney Disease Entitlement: The Social Security Amendments of 1972 (National Academy Press, 1991).

[8] D. E. Weiner , “The 2011 ESRD Prospective Payment System: Welcome to the Bundle,” American Journal of Kidney Diseases 57, no. 4 (2011): 539–541, 10.1053/j.ajkd.2011.01.007. 21333431

[9] Centers for Medicare & Medicaid Services , “End Stage Renal Disease Quality Incentive Program,” (Centers for Medicare & Medicaid Services, 2026), https://www.cms.gov/medicare/quality/end‐stage‐renal‐disease‐esrd‐quality‐incentive‐program.

[10] R. R. Quinn , N. N. Lam , P. Ravani , M. J. Oliver , P. G. Blake , and M. Tonelli , “The Advancing American Kidney Health Initiative: The Challenge of Measuring Success,” Journal of the American Society of Nephrology: JASN 33, no. 6 (2022): 1060–1062, 10.1681/ASN.2021121619. 35351817 PMC9161792

[11] N. R. Burrows , A. Koyama , and M. E. Pavkov , “Reported Cases of End‐Stage Kidney Disease ‐ United States, 2000–2019,” MMWR. Morbidity and Mortality Weekly Report 71, no. 11 (2022): 412–415, 10.15585/mmwr.mm7111a3. 35298452 PMC8942306

[12] A. Raja , S. Raja , S. B. Amin , M. Salman , B. Azeem , and L. Kumar , “Temporal Trends in Hypertension Related End Stage Renal Disease Mortality Rates: An Analysis of Gender, Race/Ethnicity, and Geographic Disparities in the United States,” Frontiers in Nephrology 3 (2023): 1339312, 10.3389/fneph.2023.1339312. 38288382 PMC10823365

[13] K. P. McCullough , H. Morgenstern , R. Saran , W. H. Herman , and B. M. Robinson , “Projecting ESRD Incidence and Prevalence in the United States Through 2030,” Journal of the American Society of Nephrology: JASN 30, no. 1 (2019): 127–135, 10.1681/ASN.2018050531. 30559143 PMC6317596

[14] E. A. Baerman , J. Kaplan , J. I. Shen , W. C. Winkelmayer , and K. F. Erickson , “Cost Barriers to More Widespread Use of Peritoneal Dialysis in the United States,” Journal of the American Society of Nephrology 33, no. 6 (2022): 1063–1072, 10.1681/ASN.2021060854. 35314456 PMC9161798

[15] A. K. Bello , I. G. Okpechi , M. A. Osman , et al., “Epidemiology of Haemodialysis Outcomes,” Nature Reviews. Nephrology 18, no. 6 (2022): 378–395, 10.1038/s41581-022-00542-7. 35194215 PMC8862002

[16] C. E. Lok , T. S. Huber , T. Lee , et al., “KDOQI Clinical Practice Guideline for Vascular Access: 2019 Update,” American Journal of Kidney Diseases 75, no. 4 (2020): S1–S164, 10.1053/j.ajkd.2019.12.001. 32778223

[17] M. Allon , “Vascular Access for Hemodialysis Patients: New Data Should Guide Decision Making,” Clinical Journal of the American Society of Nephrology 14, no. 6 (2019): 954–961, 10.2215/CJN.00490119. 30975657 PMC6556719

[18] S. van Meurs , J. Hopman , G. Hubens , et al., “Impact of Risk Factors on the Incidence of Tunneled dialysis Catheter Infections: A Systematic Review and Meta‐Analysis,” Acta Chirurgica Belgica 125, no. 1 (2025): 1–13, 10.1080/00015458.2024.2397177. 39233670

[19] V. Ramanathan , E. J. Chiu , J. T. Thomas , A. Khan , G. M. Dolson , and R. O. Darouiche , “Healthcare Costs Associated With Hemodialysis Catheter‐Related Infections: A Single‐Center Experience,” Infection Control and Hospital Epidemiology 28, no. 5 (2007): 606–609, 10.1086/513617. 17464925

[20] W. Wahood , E. Takahashi , D. Rajan , and S. Misra , “National Trends in Complications of Vascular Access for Hemodialysis and Analysis of Racial Disparities Among Patients With End‐Stage Renal Disease in the Inpatient Setting,” Kidney International Report 8, no. 6 (2023): 1162–1169, 10.1016/j.ekir.2023.03.001. PMC10239770 37284686

[21] A. Bitunguramye , G. Nkundimana , A. M. Aboubasha , et al., “Incidence, Risk Factors, Organism Types, and Outcomes of Catheter‐Related Bloodstream Infections in Hemodialysis Patients,” Cureus 16, no. 9 (2024): e69554, 10.7759/cureus.69554. 39291254 PMC11406115

[22] S. Kotwal , A. Cass , S. Coggan , et al., “Multifaceted Intervention to Reduce Haemodialysis Catheter Related Bloodstream Infections: REDUCCTION Stepped Wedge, Cluster Randomised Trial,” BMJ 377 (2022): e069634, 10.1136/bmj-2021-069634. 35414532 PMC9002320

[23] A. Al‐Balas , T. Lee , C. J. Young , J. A. Kepes , J. Barker‐Finkel , and M. Allon , “The Clinical and Economic Effect of Vascular Access Selection in Patients Initiating Hemodialysis With a Catheter,” Journal of the American Society of Nephrology 28, no. 12 (2017): 3679–3687, 10.1681/ASN.2016060707. 28710090 PMC5698057

[24] B. Lazarus , S. Kotwal , M. Gallagher , et al., “Effect of a Multifaceted Intervention on the Incidence of Hemodialysis Catheter Dysfunction in a National Stepped‐Wedge Cluster Randomized Trial,” Kidney International Report 8, no. 10 (2023): 1941–1950, 10.1016/j.ekir.2023.07.013. PMC10577327 37849996

[25] A. Al‐Balas , A. Almehmi , R. Varma , H. Al‐Balas , and M. Allon , “De Novo Central Vein Stenosis in Hemodialysis Patients Following Initial Tunneled Central Vein Catheter Placement,” Kidney360 3, no. 1 (2022): 99–102, 10.34067/KID.0005202021. 35368564 PMC8967595

[26] Z. Wang , K. Wang , and Y. Xu , “Friction Injury of the Central Vein Caused by Catheter for Hemodialysis: An In Vitro Study,” Scientific Reports 14, no. 1 (2024): 5836, 10.1038/s41598-024-56485-5. 38462667 PMC10925602

[27] M. Kächele , L. Bettac , C. Hofmann , et al., “Feasibility Analysis of Ultrasound‐Guided Placement of Tunneled Hemodialysis Catheters,” Kidney International Report 8, no. 10 (2023): 2001–2007, 10.1016/j.ekir.2023.07.038. PMC10577359 37849990

[28] J. Venegas‐Ramírez , G. A. Hernández‐Fuentes , C. S. Palomares , et al., “Vascular Access Type and Survival Outcomes in Hemodialysis Patients: A Seven‐Year Cohort Study,” Medicina (Mexico) 61, no. 4 (2025): 584, 10.3390/medicina61040584. PMC12028852 40282874

[29] T. S. Huber , S. A. Berceli , S. T. Scali , et al., “Arteriovenous Fistula Maturation, Functional Patency, and Intervention Rates,” JAMA Surgery 156, no. 12 (2021): 1111–1118, 10.1001/jamasurg.2021.4527. 34550312 PMC8459303

[30] K. Shetty , A. Kumar , R. Pararajasingham , and S. Mir , “COVID‐19 Pandemic Impact on Dialysis Access Procedures for End Stage Renal Disease Patients,” EJVES Vascular Forum 54 (2022): e29–e30, 10.1016/j.ejvsvf.2021.12.040.

[31] S. Ramanarayanan , S. Sharma , O. Swift , K. R. Laws , H. Umar , and K. Farrington , “Systematic Review and meta‐Analysis of Preoperative Interventions to Support the Maturation of Arteriovenous Fistulae in Patients With Advanced Kidney Disease,” Nephrology, Dialysis, Transplantation 38, no. 10 (2023): 2330–2339, 10.1093/ndt/gfad040. PMC10539203 36805738

[32] N. W. Kong , J. M. Kim , A. K. Krawisz , et al., “Outcomes Following Arteriovenous Fistula Creation in Medicare Beneficiaries With End‐Stage Kidney Disease,” American Journal of Cardiology 234 (2025): 79–86, 10.1016/j.amjcard.2024.10.006. 39447721 PMC11631655

[33] H. Vergara‐Pérez , R. Diaitz‐Usetxi Laplaza , P. Baliño Remiro , et al., “Comparative Outcomes of Surgical Versus Percutaneous Arteriovenous Fistulas: A Prospective Study,” Clinical Kidney Journal 18, no. 4 (2025): sfaf063, 10.1093/ckj/sfaf063. 40242052 PMC12000871

[34] E. D. Dillavou , J. F. Lucas , K. Woodside , et al., “VasQ U.S. Pivotal Study Demonstrates the Safety and Effectiveness of an External Vascular Support for Arteriovenous Fistula Creation,” Journal of Vascular Surgery 78, no. 5 (2023): 1302–1312.e3, 10.1016/j.jvs.2023.07.054. 37527689

[35] E. Russu , A. C. Munteanu , E. M. Arbănași , et al., “Out‐Patient Versus In‐Patient Arteriovenous Fistula Creation for Dialysis: Assessing Cost‐Effectiveness Alongside Clinical Implications,” Healthcare 12, no. 11 (2024): 1102, 10.3390/healthcare12111102. 38891176 PMC11171627

[36] N. S. Panse , G. E. Mina , Y. Yu , et al., “Arteriovenous Fistula Creation and Care in an Office‐Based Practice Compared With Hospital‐Based Care,” Journal of Vascular Surgery 81, no. 5 (2025): 1193–1200.e2, 10.1016/j.jvs.2025.01.002. 39800116

[37] B. Mulaney‐Topkar , V. T. Ho , M. D. Sgroi , M. Garcia‐Toca , and E. L. George , “Cost‐Effectiveness Analysis of Endovascular vs Surgical Arteriovenous Fistula Creation in the United States,” Journal of Vascular Surgery 79, no. 2 (2024): 366–381.e1, 10.1016/j.jvs.2023.11.009. 37952783

[38] C. Rognoni , M. Tozzi , and R. Tarricone , “Endovascular Versus Surgical Creation of Arteriovenous Fistula in Hemodialysis Patients: Cost‐Effectiveness and Budget Impact Analyses,” Journal of Vascular Access 22, no. 1 (2021): 48–57, 10.1177/1129729820921021. 32425096 PMC7897778

[39] R. J. Nordyke , H. Reichert , L. C. Bylsma , et al., “Costs Attributable to Arteriovenous Fistula and Arteriovenous Graft Placements in Hemodialysis Patients With Medicare Coverage,” American Journal of Nephrology 50, no. 4 (2019): 320–328, 10.1159/000502507. 31434095

[40] F. Stewart , K. Kistler , Y. Du , R. R. Singh , B. B. Dean , and S. X. Kong , “Exploring Kidney Dialysis Costs in the United States: A Scoping Review,” Journal of Medical Economics 27, no. 1 (2024): 618–625, 10.1080/13696998.2024.2342210. 38605648

[41] R. S. Kaplan and M. E. Porter , “How to Solve the Cost Crisis in Health Care,” Harvard Business Review 89, no. 9 (2011): 46–52. 21939127

[42] P. Ravani , R. Quinn , M. Oliver , et al., “Examining the Association Between Hemodialysis Access Type and Mortality: The Role of Access Complications,” Clinical Journal of the American Society of Nephrology 12, no. 6 (2017): 955–964, 10.2215/CJN.12181116. 28522650 PMC5460718

[43] A. V. Fonseca , M. G. Toledo Barros , J. C. Baptista‐Silva , J. E. Amorim , and V. Vasconcelos , “Interventions for Thrombosed Haemodialysis Arteriovenous Fistulas and Grafts,” Cochrane Database of Systematic Reviews 2, no. 2 (2024): CD013293, 10.1002/14651858.CD013293.pub2. 38353936 PMC10866196

[44] C. E. Lok , T. S. Huber , A. Orchanian‐Cheff , and D. K. Rajan , “Arteriovenous Access for Hemodialysis: A Review,” Journal of the American Medical Association 331, no. 15 (2024): 1307–1317, 10.1001/jama.2024.0535. 38497953

[45] W. S. Lindquester , R. Dhangana , and S. Warhadpande , “Bundled Medicare Payments: Trends in Utilization and Physician Payments for Dialysis Arteriovenous Fistula and Graft Maintenance Procedures From 2010 to 2018,” American Journal of Roentgenology 215, no. 4 (2020): 785–789, 10.2214/ajr.19.22675. 32783553

[46] R. Sorber , J. K. Canner , C. J. Abularrage , et al., “Quantifying the Costs of Creating and Maintaining Hemodialysis Access in an All‐Payer Rate‐Controlled Health System,” Annals of Vascular Surgery 76 (2021): 142–151, 10.1016/j.avsg.2021.05.008. 34153489 PMC8595578

[47] M. Thamer , T. C. Lee , H. Wasse , et al., “Medicare Costs Associated With Arteriovenous Fistulas Among US Hemodialysis Patients,” American Journal of Kidney Diseases 72, no. 1 (2018): 10–18, 10.1053/j.ajkd.2018.01.034. 29602630

[48] A. Ghimire , A. M. Lloyd , S. Szigety , et al., “Prospective Analysis of Arteriovenous Fistula Performance in the Context of Competing Risks,” Kidney360 6, no. 2 (2025): 272–283, 10.34067/KID.0000000650. 39560989 PMC11882251

[49] M. Atiquzzaman , B. Zhu , A. Romann , et al., “Kidney Failure Risk Equation in Vascular Access Planning: A Population‐Based Study Supporting Value in Decision Making,” Clinical Kidney Journal 17, no. 2 (2024): sfae008, 10.1093/ckj/sfae008. 38327282 PMC10847629

[50] U. Hahn Lundström , C. L. Ramspek , F. W. Dekker , et al., “Clinical Impact of the Kidney Failure Risk Equation for Vascular Access Planning,” Nephrology, Dialysis, Transplantation 39, no. 12 (2024): 2079–2087, 10.1093/ndt/gfae064. PMC11648961 38486367

[51] K. Kuningas , S. Stringer , P. Cockwell , A. Khawaja , and N. Inston , “Is There a Role of the Kidney Failure Risk Equation in Optimizing Timing of Vascular Access Creation in Pre‐Dialysis Patients?” Journal of Vascular Access 24, no. 6 (2023): 1305–1313, 10.1177/11297298221084799. 35343295

[52] J. Li , H. Lu , Z. Xie , Q. Li , and H. Shi , “Outcomes of Arteriovenous Graft vs. Fistula for Haemodialysis Access in the Elderly: A Systematic Review and Meta‐Analysis,” Experimental and Therapeutic Medicine 26, no. 2 (2023): 399, 10.3892/etm.2023.12098. 37522056 PMC10375446

[53] B. Edgar , C. Jones , E. Aitken , et al., “What Are the Reported Procedural Costs of Vascular Access Surgery?” Journal of Vascular Access 26 (2024): 11297298241284737, 10.1177/11297298241284737. 39344914

[54] Sidecar Health , “Cost of AV Fistula Creation for Dialysis,” (Sidecar Health, 2025), https://cost.sidecarhealth.com/n/av‐fistula‐creation‐for‐dialysis‐cost.

[55] R. J. Halbert , G. Nicholson , R. J. Nordyke , A. Pilgrim , and L. Niklason , “Patency of ePTFE Arteriovenous Graft Placements in Hemodialysis Patients: Systematic Literature Review and Meta‐Analysis,” Kidney360 1, no. 12 (2020): 1437–1446, 10.34067/KID.0003502020. 35372887 PMC8815525

[56] A. A. Al‐Jaishi , M. J. Oliver , S. M. Thomas , et al., “Patency Rates of the Arteriovenous Fistula for Hemodialysis: A Systematic Review and Meta‐Analysis,” American Journal of Kidney Diseases 63, no. 3 (2014): 464–478, 10.1053/j.ajkd.2013.08.023. 24183112

[57] N. S. Roetker , H. Guo , D. R. Ramey , C. J. McMullan , G. B. Atkins , and J. B. Wetmore , “Hemodialysis Access Type and Access Patency Loss: An Observational Cohort Study,” Kidney Medicine 5, no. 1 (2023): 100567, 10.1016/j.xkme.2022.100567. 36636202 PMC9829958

[58] B. Lyu , M. R. Chan , A. S. Yevzlin , and B. C. Astor , “Catheter Dependence After Arteriovenous Fistula or Graft Placement Among Elderly Patients on Hemodialysis,” American Journal of Kidney Diseases 78, no. 3 (2021): 399–408.e1, 10.1053/j.ajkd.2020.12.019. 33582176

[59] Centers for Medicare & Medicaid Services . “Medicare NCCI Policy Manual,” (Centers for Medicare & Medicaid Services, 2025), https://www.cms.gov/medicare/coding‐billing/national‐correct‐coding‐initiative‐ncci‐edits/medicare‐ncci‐policy‐manual.

[60] R. Fluck and M. Kumwenda , “Renal Association Clinical Practice Guideline on Vascular Access for Haemodialysis,” Nephron. Clinical Practice 118, no. Suppl 1 (2011): c225–c240, 10.1159/000328071. 21555898

[61] B. Fila , “Quality Indicators of Vascular Access Procedures for Hemodialysis,” International Urology and Nephrology 53, no. 3 (2021): 497–504, 10.1007/s11255-020-02609-5. 32869172

[62] T. M. Vesely , G. Beathard , S. Ash , J. Hoggard , and D. Schon , “Classification of Complications Associated With Hemodialysis Vascular Access Procedures: A Position Statement From the American Society of Diagnostic and Interventional Nephrology,” Seminars in Dialysis 20, no. 4 (2007): 359–364. 17635830 10.1111/j.1525-139X.2007.00318.x

[63] T. Coleman , A. Dasgupta , and C. G. Carsten III , “Preparing a Dialysis Patient,” Seminars in Vascular Surgery 37, no. 4 (2024): 364–368. 39675843 10.1053/j.semvascsurg.2024.10.004

[64] A. Lubitz and K. Woo , “Choice of Dialysis Access: Catheter, Peritoneal, or Hemodialysis,” Seminars in Vascular Surgery 37, no. 4 (2024): 369–374, 10.1053/j.semvascsurg.2024.09.003. 39675844

[65] B. Fila , R. Roca‐Tey , J. Malik , et al., “Quality Assessment of Vascular Access Procedures for Hemodialysis: A Position Paper of the Vascular Access Society Based on the Analysis of Existing Guidelines,” Journal of Vascular Access 21, no. 2 (2020): 148–153, 10.1177/1129729819848624. 31106700

[66] C. Bleustein , D. B. Rothschild , A. Valen , E. Valatis , L. Schweitzer , and R. Jones , “Wait Times, Patient Satisfaction Scores, and the Perception of Care,” American Journal of Managed Care 20, no. 5 (2014): 393–400. 25181568

[67] R. D. Malgor , J. M. Baker , E. A. Malgor , and J. Blebea , “Endovascular Experience at an Academic Office‐Based Procedure Center,” Vascular 31, no. 2 (2023): 226–233, 10.1177/17085381211059651. 35331076

[68] R. Margulis , D. J. Pedulla , A. L. Bromberg , and C. Choice , “Evaluation of the Safety of Arteriovenous Fistula Creation Surgery in Ambulatory Versus Inpatient Hospital Setting,” Saudi Journal of Kidney Diseases and Transplantation 30, no. 6 (2019): 1295–1299, 10.4103/1319-2442.275473. 31929276

[69] Medicare and Medicaid Programs: Hospital Outpatient Prospective Payment and Ambulatory Surgical Center Payment Systems; Quality Reporting Programs; Overall Hospital Quality Star Ratings; and Hospital Price Transparency (Federal Register, 2025. Accessed July 23, 2025) https://www.federalregister.gov/documents/2025/07/17/2025‐13360/medicare‐and‐medicaid‐programs‐hospital‐outpatient‐prospective‐payment‐and‐ambulatory‐surgical.

[70] C. W. Hicks , M. Bronsert , K. E. Hammermeister , et. al, “Temporal Trends, Determinants, and Outcomes of Inpatient Versus Outpatient Arteriovenous Fistula Operations,” Annals of Vascular Surgery 46 (2018): 65–74.e1. 28887240 10.1016/j.avsg.2017.07.032

[71] K. S. Brimble , C. G. Rabbat , D. Schiff , and A. J. Ingram , “The Clinical Utility of Doppler Ultrasound Prior to Arteriovenous Fistula Creation,” Seminars in Dialysis 14, no. 5 (2001): 314–317, 10.1046/j.1525-139x.2001.00077.x. 11679094

[72] E. Mateos Torres , S. Collado Nieto , H. Cao Baduell , M. Lacambra Peñart , A. Velescu , and A. Clará Velasco , “Utility of Doppler Ultrasound in the Preoperative Evaluation of the First Vascular Access for Haemodialysis,” Nefrología 39, no. 5 (2019): 539–544, 10.1016/j.nefro.2019.02.012. 31377029

[73] I. Aragoncillo Sauco , J. M. Ligero Ramos , A. Vega Martínez , et al., “Clinic of Vascular Access: Results After Implementing a Multidisciplinary Approach Adding Routine Doppler Ultrasound,” Nefrología (English Edition) 38, no. 6 (2018): 616–621, 10.1016/j.nefroe.2018.11.005. 29903522

[74] M. Thomas , C. Nesbitt , M. Ghouri , and M. Hansrani , “Maintenance of Hemodialysis Vascular Access and Prevention of Access Dysfunction: A Review,” Annals of Vascular Surgery 43 (2017): 318–327, 10.1016/j.avsg.2017.02.014. 28478166

[75] Y. Etkin , S. Talathi , A. Rao , et al., “The Role of Duplex Ultrasound in Assessing AVF Maturation,” Annals of Vascular Surgery 72 (2021): 315–320, 10.1016/j.avsg.2020.10.006. 33227470

[76] A. Cansu , M. Soyturk , M. H. Ozturk , S. Kul , Z. Pulathan , and H. Dinc , “Diagnostic Value of Color Doppler Ultrasonography and MDCT Angiography in Complications of Hemodialysis Fistulas and Grafts,” European Journal of Radiology 82, no. 9 (2013): 1436–1443, 10.1016/j.ejrad.2013.03.015. 23660569

[77] J. J. Sands , L. M. Ferrell , and M. A. Perry , “The Role of Color Flow Doppler Ultrasound in dialysis Access,” Seminars in Nephrology 22, no. 3 (2002): 195–201, 10.1053/snep.2002.31705. 12012305

[78] A. Mudoni , F. Caccetta , M. Caroppo , et al., “Echo Color Doppler Ultrasound: A Valuable Diagnostic Tool in the Assessment of Arteriovenous Fistula in Hemodialysis Patients,” Journal of Vascular Access 17, no. 5 (2016): 446–452, 10.5301/jva.5000588. 27470250

[79] N. R. Dossabhoy , S. J. Ram , R. Nassar , J. Work , J. M. Eason , and W. D. Paulson , “Stenosis Surveillance of Hemodialysis Grafts by Duplex Ultrasound Reduces Hospitalizations and Cost of Care,” Seminars in Dialysis 18, no. 6 (2005): 550–557, 10.1111/j.1525-139X.2005.00102.x. 16398720

[80] J. R. A. Lopes , A. L. d. B. Marques , and J. A. Correa , “Randomised Clinical Study of the Impact of Routine Preoperative Doppler Ultrasound for the Outcome of Autologous Arteriovenous Fistulas for Haemodialysis,” Journal of Vascular Access 22, no. 1 (2021): 107–114, 10.1177/1129729820927273. 32519569 PMC7897791

[81] J. Kott , N. Reichek , J. Butler , L. Arbeit , and S. K. Mallipattu , “Cardiac Imaging in Dialysis Patients,” Kidney Medicine 2, no. 5 (2020): 629–638, 10.1016/j.xkme.2020.05.010. 33094277 PMC7568079

[82] L. Guo , Y. Ji , T. Sun , et al., “Management of Chronic Heart Failure in Dialysis Patients: A Challenging but Rewarding Path,” Reviews in Cardiovascular Medicine 25, no. 6 (2024): 232, 10.31083/j.rcm2506232. 39076321 PMC11270084

[83] J. A. Spertus and P. G. Jones , “Development and Validation of a Short Version of the Kansas City Cardiomyopathy Questionnaire,” Circulation. Cardiovascular Quality and Outcomes 8, no. 5 (2015): 469–476, 10.1161/CIRCOUTCOMES.115.001958. 26307129 PMC4885562

[84] N. Klomjit , A. G. Kattah , and W. Cheungpasitporn , “The Cost‐Effectiveness of Peritoneal Dialysis Is Superior to Hemodialysis: Updated Evidence From a More Precise Model,” Kidney Medicine 3, no. 1 (2020): 15–17, 10.1016/j.xkme.2020.12.003. 33605939 PMC7873827

[85] K. L. Johansen , G. M. Chertow , D. T. Gilbertson , et al., “US Renal Data System 2022 Annual Data Report: Epidemiology of Kidney Disease in the United States,” American Journal of Kidney Diseases 81, no. 3 Suppl1 (2023): A8–A11, 10.1053/j.ajkd.2022.12.001. 36822739 PMC10807034

[86] J. G. Heaf and S. Wehberg , “Relative Survival of Peritoneal Dialysis and Haemodialysis Patients: Effect of Cohort and Mode of Dialysis Initiation,” PLoS ONE 9, no. 3 (2014): e90119, 10.1371/journal.pone.0090119. 24614569 PMC3948631

[87] J. M. Kaplan , J. Niu , V. Ho , W. C. Winkelmayer , and K. F. Erickson , “A Comparison of US Medicare Expenditures for Hemodialysis and Peritoneal Dialysis,” Journal of the American Society of Nephrology 33, no. 11 (2022): 2059–2070, 10.1681/ASN.2022020221. 35981764 PMC9678042

[88] R. Figueiredo , M. Relvas , H. Diniz , et al., “The Impact of Multidisciplinary Vascular Access Team Creation in Incident Dialysis Patients: A Retrospective Case‐Control Study,” Journal of Vascular Access 27 (2025): 11297298251344069, 10.1177/11297298251344069. 40444768

[89] C. E. Holden‐Wingate , L. R. Holden‐Wingate , A. Hussain , et al., “Team‐Based Approach to Arteriovenous Access Management,” Seminars in Vascular Surgery 38 (2025): 404–411, 10.1053/j.semvascsurg.2025.08.002. 41386915 PMC13247990

[90] M. Matsui , M. Kokubu , M. Nishimoto , et al., “Association of eGFR Slopes With Cardiorenal Outcomes in Chronic Kidney Disease Patients Before and After Multidisciplinary Education,” Research Square Preprint (2024), 10.21203/rs.3.rs-4398000/v1. PMC12995348 41591964

[91] H. T. Hsu , Y. C. Chiang , Y. H. Lai , L. Y. Lin , H. F. Hsieh , and J. L. Chen , “Effectiveness of Multidisciplinary Care for Chronic Kidney Disease: A Systematic Review,” Worldviews on Evidence‐Based Nursing 18, no. 1 (2021): 33–41, 10.1111/wvn.12483. 33247619

[92] Y. Imamura , Y. Takahashi , S. Uchida , et al., “Effect of Multidisciplinary Care of dialysis Initiation for Outpatients With Chronic Kidney Disease,” International Urology and Nephrology 53, no. 7 (2021): 1435–1444, 10.1007/s11255-021-02787-w. 33590452

[93] A. Theeranut , N. Methakanjanasak , P. Surit , et al., “Can a Multidisciplinary Approach Slow Renal Progression in CKD Patients?” International Journal of Medical Sciences 18, no. 9 (2021): 1975–1979, 10.7150/ijms.53189. 33850467 PMC8040396

[94] R. Mishler , J. J. Sands , N. J. Ofsthun , M. Teng , D. Schon , and J. M. Lazarus , “Dedicated Outpatient Vascular Access Center Decreases Hospitalization and Missed Outpatient dialysis Treatments,” Kidney International 69, no. 2 (2006): 393–398, 10.1038/sj.ki.5000066. 16408132

[95] P. Rios , L. Sola , A. Ferreiro , et al., “Adherence to Multidisciplinary Care in a Prospective Chronic Kidney Disease Cohort Is Associated With Better Outcomes,” PLoS ONE 17, no. 10 (2022): e0266617, 10.1371/journal.pone.0266617. 36240220 PMC9565398

[96] S. Piri , “Pay‐For‐Performance Programs Effectiveness in Healthcare: The Case of the End‐Stage Renal Disease Quality Incentive Program,” European Journal of Health Economics 25, no. 2 (2024): 221–236, 10.1007/s10198-023-01582-x. 36966480

